# Motivational Profiles of Adolescent Gaming and Their Associations with Psychopathology and Well-Being

**DOI:** 10.1007/s10964-026-02414-w

**Published:** 2026-09-24

**Authors:** Jennipher Jildenstål, Sabina Kapetanovic, Sevtap Gurdal

**Affiliations:** 1 Department of Psychology, Pedagogy and Sociology, University West, Trollhättan, Sweden; 2 Department of Psychology, University of Inland, Lillehammer, Norway

**Keywords:** Adolescents, Gaming Motives, Motivational Profiles, Psychopathology, Well-Being

## Abstract

Digital gaming is a common leisure activity among adolescents, yet its relationship with mental health remains poorly understood. Emerging evidence suggests that gaming motivations may be more informative than gaming behavior per se for understanding adolescent psychological outcomes. The present study examined associations between gaming motives and mental health and identified distinct motivational profiles among adolescent gamers. Participants were Swedish adolescents aged 14–17 years (*n* = 418; *62.2*% boys). Gaming motives were assessed across seven domains, and mental health was operationalized across both dimensions of the dual-continua model: psychopathology, indexed by internalizing and externalizing problems, and positive mental health, indexed by subjective well-being. Latent profile analysis identified four motivational profiles: low-motive gamers, broadly motivated gamers, competition-oriented gamers, and highly motivated gamers. Avoidance-oriented motives (escape, coping, fantasy) were associated with lower well-being and higher internalizing and externalizing problems, whereas competence-related motives (competition, skill development) were associated with more favorable well-being. Two profiles represented potential clinical risk groups: broadly motivated gamers reported the highest internalizing problems, and highly motivated gamers the highest externalizing problems. These findings suggest that assessing gaming motivations and not merely gaming frequency, may improve identification of at-risk adolescents and inform more targeted clinical screening and intervention.

**Supplementary Information:**

The online version contains supplementary material available at https://doi.org/10.1007/s10964-026-02414-w.

## Introduction

Digital gaming is a central part of many adolescents’ everyday lives, providing contexts for social interaction, competition, and recreation (Paulus et al., 2018). Although gaming is harmless for many adolescents, it may be associated with distress for some (Wartberg et al., 2017). Research has often focused on time spent gaming, but gaming duration alone shows weak or inconsistent associations with adolescent psychopathology and well-being (Ballou et al., 2025). Consequently, the focus has moved from *how much* adolescent game to *why* they do (Király et al., 2017). Gaming motives reflect the underlying psychological functions that gaming serves for the individual (Király et al., 2017), including social interaction, competition, recreation, escape, coping, skill development, and fantasy (Demetrovics et al., 2011).

Emerging evidence suggests that motives are differentially associated with adolescent psychopathology and well-being. Escape and coping motives have been linked to depressive symptoms (Wartberg et al., 2017; Wichstrøm et al., 2019), whereas social and recreational motives have been associated with higher well-being and fewer psychological difficulties (Stevens et al., 2021). However, motives rarely occur in isolation, and relatively little research has examined how combinations of motives cluster within adolescent gamers and relate to both psychopathology and well-being. The present study addresses this gap by using latent profile analysis to identify motivational profiles and examine their associations with internalizing problems, externalizing problems, and subjective well-being.

### Adolescence as a Critical Developmental Period

Adolescence is a developmental stage defined by rapid biological, psychological, and social changes (Sawyer et al., 2018). Spanning approximately ages 10 to 19, it involves accelerated physical maturation alongside continued brain development, as well as ongoing development in emotion regulation, identity formation, and peer relationships (Dahl et al., 2018). Academic demands, changing family dynamics, and expanding social roles introduce new developmental challenges, making adolescence a period of both heightened vulnerability and considerable opportunity (Dahl et al., 2018; Sawyer et al., 2018). Beyond its developmental significance, adolescence carries considerable implications for mental health, as many psychological difficulties first emerge during this period (Solmi et al., 2022). Psychopathology within adolescence is commonly organized along two broad dimensions: internalizing problems, reflecting emotional difficulties directed inward, and externalizing problems, reflecting behavioral difficulties directed outward (Achenbach & Rescorla, 2004; Plenty et al., 2021). Internalizing symptoms such as anxiety and depression rise markedly during adolescence, with growing gender differences in prevalence (Solmi et al., 2022), while externalizing behaviors tend to emerge earlier and show greater stability across this period (Achenbach & Rescorla, 2004). Although interrelated, these dimensions reflect distinct aspects of psychopathology associated with different developmental processes and risk factors (Mason Stephens et al., 2023).

Focusing solely on psychopathology, however, provides an incomplete picture of adolescent mental health. Thus, mental health is not merely the absence of disorder but encompasses the presence of positive functioning and emotional experience. Dual-continua models formalize this distinction, proposing that psychopathology and well-being represent related but separable dimensions (Keyes, 2005), and suggesting that low symptom levels do not automatically imply high well-being, and vice versa. Well-being is commonly conceptualized as comprising a hedonic component including positive affective states such as optimismallagher et al., and positive mood, and a eudaimonic component, emphasizing autonomy, competence, resilience, and positive relationships (De Cates et al., 2015). Higher levels of well-being predict a range of adaptive outcomes, including better emotion regulation, resilience, academic engagement, and social functioning, even after controlling for internalizing and externalizing symptoms (Collado-Soler et al., 2023), underscoring its role as a core psychological resource for healthy adolescent development.

### Adolescent Gaming

Against the backdrop of these mental health considerations, it is important to examine the contexts in which adolescents spend their free time. One of the most prominent of these contexts is digital gaming. While dictionary definitions emphasize its technological dimension (Cambridge Dictionary, n.d.), research conceptualizes gaming more broadly as an interactive, goal-oriented, and socially embedded activity that can fulfill a range of psychological and emotional functions (Caroux et al., 2015). Gaming is therefore not simply a behavior to measure, but a psychological space to understand.. In Sweden, approximately 90% of boys and 70% of girls aged 13–18 game regularly (Andersson, 2025). Beyond entertainment, gaming provides opportunities for social interaction, competition, immersion, and emotional experience (Bäcklund et al.,^2025^). Online multiplayer environments enable communication and shared goal pursuit, creating socially structured contexts in which players negotiate roles and status (Sirola et al., 2021), and through avatars and in-game group membership, adolescents may explore aspects of identity and social positioning within flexible digital spaces (Kaye et al., 2017). These diverse functions make the relationship between gaming and mental health difficult to evaluate. Research consistently shows that its effects are far from uniform: moderate, well-regulated gaming is generally not linked to poorer psychological functioning and may even relate positively to well-being, while evidence regarding externalizing behaviors is more mixed, with associations with aggression largely disappearing when prior aggression is controlled (Johannes et al., 2021; Prescott et al., 2018). Ultimately, the key question is not whether gaming is good or bad, but when, how, and for whom it matters.

### Gaming Motives: Uses and Gratification Theory (UGT)

Most research on adolescent gaming has focused on time spent gaming, with less attention to how underlying motivations interact with gaming frequency in shaping mental health outcomes. The Motives for Online Gaming Questionnaire (MOGQ) was developed to assess seven distinct dimensions of gaming motivation (Demetrovics et al., 2011). These can be broadly grouped into engagement-oriented motives, including social (i.e., playing to interact with others and build relationships), competition (i.e., playing to win and outperform others), recreation (i.e., playing for enjoyment, fun, and leisure) and skill development (i.e., playing to improve strategic, cognitive, or motor abilities); and avoidance-oriented motives including fantasy (i.e., playing to experience alternative identifies or immerse oneself in fictional worlds), escape (i.e., playing to avoid real-life problems or negative emotions), and coping (i.e., playing to reduce stress or regulate mood). In line with Uses and Gratifications theory, which assumes that individuals actively select media to satisfy specific psychological needs, the gaming motives taxonomy conceptualizes gaming as a goal-directed and functional activity rather than uniform behavior (Katz et al., 1973). The integration of gaming motives and uses and gratification theory enhances explanatory clarity by specifying the psychological needs underlying each identified gaming motive, thereby situating individual motives within a broader framework of goal-directed gratification-seeking. Each gaming motivation can be understood as a specific gratification-seeking pattern.

The social motive reflects the pursuit of belonging and peer integration. The recreation motive reflects pure entertainment and hedonic enjoyment. The competition and skill development motives reflect achieve and competence-based gratifications. Gaming offers quantifiable feedback systems, ranking, levels, and performance statistics, that few offline environments provide with equal immediacy and clarity (Koivisto & Hamari, 2019). The fantasy motive reflects identity experimentation and self-expansion, allowing adolescents to explore alternative roles and selves in low-risk environments (Demetrovics et al., 2011). However, the theoretical differentiation between recreationally gratifying and psychologically regulatory motives becomes particularly salient when examining the escape and coping dimensions (King & Delfabbro, 2020). Escape involves using gaming to avoid real-life problems, whereas coping refers to mood regulation and stress reduction (Demetrovics et al., 2011). Within uses and gratification theory, these fall under affective and tension-release gratifications. The key argument is that, when gaming shifts from being recreationally gratifying to being psychologically regulatory, its functional role changes. In other words, not all motives carry equal developmental implications. Gaming motivated by recreation, skill development, or social connection may be adaptive, developmentally normative, and even beneficial (Johannes et al., 2021). In contrast, gaming motivated by escape or coping may reflect attempts to regulate stress or negative emotions. Importantly, such motives do not necessarily imply maladaptive functioning, as adolescents may differ in both their levels of underlying distress and the effectiveness of their existing coping resources. For some individuals, gaming may serve as a temporary and adaptive form of recovery or emotional regulation, whereas for others it may become increasingly avoidant or interfere with the development of other coping strategies (King & Delfabbro, 2020). The integration of gaming motives and uses and gratification theory may help explain why similar levels of gaming involvement are associated with different outcomes depending on whether adolescents game for recreation, mastery, belonging, or emotional regulation (Ballou et al., 2025).

Research further suggests that gaming motives may function not only as descriptive categories but also as indicators of differential psychological risk. Avoidance-oriented motives, such as escape and coping, have consistently been associated with higher levels of psychological distress and problematic engagement. Adolescents who report gaming primarily to escape from real-life problems are more likely to experience depressive and psychosomatic symptoms (Hellström et al., 2015), and escape and coping motives may mediate the association between difficulties and problematic gaming behavior (Király et al., 2017; Bäcklund et al., 2025). These findings support the notion that gaming can sometimes function as a compensatory strategy for regulating negative emotions rather than as a purely recreational activity. In contrast, engagement-oriented motives such as social interaction, competition, and recreation tend to show weaker or non-significant associations with psychological distress and may reflect more socially embedded forms of gaming. For example, socially oriented gaming has been linked to increased peer connectedness and social capital over time (Kim et al., 2022), while recreational motives are generally associated with gaming as a leisure activity rather than as a coping mechanism (Hellström et al., 2015).

### Person-Centered Approaches to Gaming Motivation

Notably, adolescents rarely engage in gaming for a single reason. Instead, multiple motives typically co-occur within individuals. Consequently, recent research has increasingly adopted person-centered approaches to examine how combinations of motives cluster within individuals. Studies using latent profile analysis have identified distinct motivational profiles among gamers that differ in levels of engagement, problematic gaming tendencies, and psychological well-being (Kim et al., 2022; Meriläinen et al., 2023). In a large sample of Finnish adolescents aged 11–14, identified three profiles: escapist gamers, characterized by elevated escape motives and the highest levels of internalizing symptoms and academic difficulties; achiever gamers, defined by prominent competence and mastery motives with moderate well-being outcomes; and recreational gamers, who gamed primarily for enjoyment and showed the most favorable well-being across all indicators (Meriläinen et al., 2023). Similarly, four profiles was identified in a sample of Korean online gamers: a highly motivated-dissatisfied profile, characterized by high escape and fantasy motives alongside low recreational motivation, which showed the most pathological outcomes including elevated Internet gaming disorder symptoms, neuroticism, and impulsivity; a highly motivated-satisfied profile reflecting high engagement with positive psychological functioning; and two casual gamer profiles distinguished by moderate and low overall motivation respectively (Kim et al., 2022). Corroborating these findings,a longitudinally study demonstrated that adolescents in “Escapers”- profile showed significantly elevated risk for psychopathology and social withdrawal relative to both recreational and achiever profiles, whereas recreational gamers consistently showed the most adaptive psychosocial outcomes (Wang et al., 2021). Taken together, these findings suggest that avoidance-oriented profiles are consistently associated with higher levels of psychological distress, whereas competence- and recreation-oriented profiles are linked to more favorable well-being. However, research examining motivational profiles in adolescent samples across both psychopathology and positive well-being remains limited.

## Current Study

Despite growing evidence that specific gaming motives are differentially associated with adolescent psychopathology and well-being, less is known about how these motives co-occur within individuals and form distinct motivational profiles. The present study therefore examined motivational profiles among adolescent gamers and their associations with internalizing problems, externalizing problems, and subjective well-being. Based on the theoretical framework and prior empirical evidence, four hypotheses were formulated. It was hypothesized that distinct avoidance-oriented and engagement-oriented motivational profiles would emerge (Hypothesis 1); that adolescents in avoidance-oriented profiles would report higher levels of internalizing problems (Hypothesis 2) and externalizing problems (Hypothesis 3); and that adolescents in engagement-oriented profiles would report higher subjective well-being (Hypothesis 4).

## Methods

### Participants

#### Total Sample

The study was conducted as part of the Gamepsy-9 project, which examines associations between adolescents’ mental health and digital media use. The project included 716 Swedish mid-adolescents (age 14–17), of whom 347 (48.5%) identified as female, 352 (49.3%) as male, 9 (1.3%) reported another gender identity, and 3 participants (0.4%) chose not to disclose their gender. Most participants were born in Sweden (83.7%), although some reported being born in other Nordic countries (0.7%), in other European countries (4.8%), or outside Europe (10.0%), a very small group reported other birth countries (0.8%). Regarding parental background, most mothers (66.9%) and fathers (65.9%) were born in Sweden. Smaller proportions were born in other Nordic countries (mothers 1.0%, fathers 2.1%), elsewhere in Europe (mothers 9.7%, fathers 8.8%), or outside Europe (mothers 21.0%, fathers 21.1%). A very small proportion reported other birth countries (mothers 1.4%, fathers 2.1%). Subjective socioeconomic status (SES) was assessed via self-report on a 5-point scale comparing the family’s financial situation to peers. The average response indicated a generally average to slightly above-average SES (*M* = 3.0, *SD* = 0.81).

Because the aim of the study was to examine gaming motives, analyses were restricted to adolescents who reported playing digital games engage in gaming. Participants who indicated that they never play digital games were therefore excluded from the analytical sample. Applying this inclusion criterion resulted in a final analytical sample of *N* = 484 adolescents. Within the analytical sample, 301 participants (62.2%) identified as boys and 183 participants (37.8%) as girls. Participants were aged 14–17 years (*M* = 15.85, *SD* = 0.45) representing mid-adolescence.

#### Attrition Analysis

Preliminary comparisons were conducted to examine whether the inclusion criterion introduced systematic differences between included and excluded participants across demographic variables (gender, age, country of birth, parental country of birth, and perceived socioeconomic status). A Chi-square test revealed significant differences in gender χ²(3, *N* = 712) = 71.90, *p* < .001, with excluded participants more likely to be girls (73.6%) than those included in the analytic sample (39.2%), whereas boys were more often included (59.2%). Significant differences were also found for country of birth, χ²(4, *N* = 712) = 32.27, *p* < .001, mothers´ country of birth, χ²(4, *N* = 714) = 16.50, *p* = .002, and fathers’ country of birth, χ²(4, *N* = 712) = 14.14, *p* = .007. Participants born outside Europe, and those with parents born outside Europe, were more likely to be excluded. No significant difference was found for perceived socioeconomic status, χ²(4, *N* = 713) = 6.55, *p* = .162. An independent samples t-test showed small but significant age difference t(703) = 2.51, *p* = .012, with excluded participants being slightly older on average.

### Procedure

A web-based questionnaire was administered to assess adolescents’ mental health, social media use, and gaming behaviors. Data were collected in collaboration with 14 secondary schools and two high schools in western Sweden. School principals and staff were informed about the study and approved participation. Information about the study was then provided to students and parents through a video presentation and written materials. Approximately one week later, members of the research team visited the schools and administrated the questionnaire.

Data collection took place during school hours in classroom setting and took approximately 60 min. At the beginning of the session, members of the research group provided a verbal explanation of the study, including its purpose, content, and ethical principles such as voluntary participation and the right to withdraw at any time without negative consequences. Students who chose to participate signed an informed consent form before completing the questionnaire. Participation was voluntary, and students who did not wish to take part were asked to work quietly on other school-related tasks in the classroom to minimize disruption. One teacher was present during the session to provide practical support if needed. Each participant was assigned an anonymized identification code by the research group to ensure that no personally identifying information was linked to the survey responses.

The study was conducted in accordance with the ethical principles of the Declaration of Helsinki and received ethical approval from the Swedish regional ethical review authority on June 4th, 2024 (ref. 2024-02585-01) and July 30th, 2025 (ref. 2025-04464-02).

According to Swedish ethical legislation, parental consent is not required for adolescents aged 15 and above. However, as a small number of participants in the participating classes had not yet reached the age of 15, written parental consent was obtained for these individuals in addition to their own informed consent.

### Measures

#### Demographical Variables

Participants reported their age by selecting one of the predefined response options (14, 15, 16, or 17 years). Gender was assessed with the question “Are you…?” with response options: girl, boy, another gender identity, and prefer not to answer. Responses indicating another gender identity or prefer not to answer were treated as missing in the binary analyses due to the very small number of cases in these categories, which did not allow meaningful statistical comparisons.

#### Gaming Motives

Gaming motives were assessed using the Motives for Online Gaming Questionnaire (MOGQ; Demetrovics et al., 2011), a self-report instrument developed to measure the psychological motivations underlying engagement in online gaming. The Swedish version of the MOGQ has been translated and validated in a Swedish sample, demonstrating good reliability and support for the original seven-factor structure (Bäcklund et al., 2024). The MOGQ consists of 27 items rated on a 5-point numerical scale ranging from 1 (almost never/never) to 5 (almost always/always). The instrument measures seven distinct motivational dimensions: social (playing to interact and build relationships with others), escape (playing to avoid real-life problems or negative emotions), competition (playing to compete and achieve status), coping (playing to reduce stress or regulate mood), skill development (playing to improve coordination, concentration or strategic thinking), fantasy (playing to experience alternative identities or virtual worlds) and recreation (playing for fun and enjoyment). Subscales scores were computed by summing the items belonging to each motivational domain, with higher scores indicating stronger endorsement of that specific gaming motive. The internal consistency of MOGQ subscales has been shown to be good to excellent: social (α = 0.85), escape (α = 0.92), competition (α = 0.87), coping (α = 0.80), skill development (α = 0.93), recreation (α = 0.78), and fantasy (α = 0.88).

#### **Psychopathology**

Internalizing and externalizing problems were assessed using the Brief Problem Monitor – Youth (BPM-Y; Achenbach et al., 2011), a brief and reliable measure derived from the Achenbach System of Empirically Based Assessment (ASEBA; Achenbach & Rescorla, 2004). The BPM-Y is designed for youth aged 6–18 years, with participants rating how well each statement described them over the past six months on a three-point scale (0 = not true, 1 = somewhat or sometimes true, 2 = very true or often true). Two of the three empirically derived subscales were used in the present study, with item responses summed to create continuous composite scores; higher scores indicate greater difficulties. The internalizing problems scale (6 items) captures anxiety, depressive mood, loneliness, and excessive worry, and demonstrated good internal consistency in the present sample (Cronbach’s α = 0.84). The externalizing problems scale originally comprises 7 items reflecting rule-breaking, aggression, and oppositional behavior. One item (“I stand my ground and disagree with others”) demonstrated a weak corrected item-total correlation and raised concerns regarding face validity; its removal improved internal consistency from α = 0.57 to α = 0.67. This item was therefore excluded from the final scale, consistent with established psychometric practice and prior research noting variability in the BPM-Y’s psychometric properties across samples (Geibel et al., 2016). The validity and reliability of Swedish ASEBA instruments in youth samples have been supported in previous research (Skarphedinsson et al., 2021).

#### Subjective Well-Being

Subjective well-being was assessed using the Swedish version of Short Warwick Edinburgh Mental Well-Being Scale (SWEMWBS) (Haver et al., 2015). The scale consists of seven positively worded items measuring aspects of psychological functioning and emotional well-being over the past two weeks. Items cover optimism about the future, feeling useful, feeling relaxed, dealing with problems well, thinking clearly, feeling close to others, and being able to make up one´s own mind (Stewart-Brown et al., 2009). Responses are rated on a 5-point numerical scale ranging from 1 (never) to 5 (always). Item scores were summed to create a total well-being score, with higher scores indicating greater mental well-being. The analysis indicated good internal consistency (α = 0.86).

### Data Analysis

Data analysis was performed using the statistical software IBM SPSS statistics (version 28), and Mplus (Version 9) (IBM Corp., 2021). Means, standard deviations, minimum values, and maximum values were calculated to provide an overview of central tendency and dispersion (Table 1). To test the psychometric properties of the seven-factor structure of the MOGQ, a confirmatory factor analysis (CFA) with maximum likelihood with robust standard errors (MLR) was estimated. The model showed somewhat mixed but generally acceptable fit, χ²(325) = 1,009.41, *p* < .001, CFI = 0.908, TLI = 0.893, RMSEA = 0.066, 90% CI [0.061, 0.071], and SRMR = 0.063. Although the CFI, RMSEA, and SRMR supported an acceptable approximation to the data (Hair et al., 2019), the TLI was slightly below 0.90, indicating some remaining model misfit. All standardized factor loadings were positive and statistically significant (*p* < .001), ranging from 0.635 to 0.800 for Social, 0.817 to 0.914 for Escape, 0.674 to 0.883 for Competition, 0.636 to 0.812 for Coping, 0.857 to 0.889 for Skill Development, 0.685 to 0.855 for Fantasy, and 0.558 to 0.823 for Recreation. Full CFA results are presented in Supplementary Table S1.

Latent profile analysis (LPA) was conducted to identify subgroups of adolescents based on seven gaming motivation indicators (social, escape, competition, coping, skill development, fantasy and recreation). LPA is a person-centered statistical approach that aims to identify relatively homogenous groups of individuals who share similar response patterns across continuous variables (Collins & Lanza, 2010). The analysis was conducted stepwise, by following common recommendations in mixture modeling research (Nylund et al., 2007). First, a one-profile model was estimated. This model assumes that all individuals belong to a single homogenous group and serves as a baseline comparison. Next, models with increasing numbers of profiles were estimated (two-, three-, four-, five-, and six-profile solutions). All models were estimated using maximum likelihood with robust standard errors (MLR). This estimator was chosen because it provides parameters estimated that are robust to moderate deviations from normality and allows missing data to be handled using full information maximum likelihood (FIML), under the assumption that data are missing at random (MAR) (Enders, 2010). All models were estimated under the assumption of local independence, meaning that associations among the motivation indicators were assumed to be explained by latent profile memberships rather than direct correlations between the indicators (Collins & Lanza, 2010). To reduce the risk of local maxima, all LPA models were estimated using 100 initial-stage and 20 final-stage random starts. Consistent with recommendations to evaluate the stability of mixture-model solutions (Spurk et al., 2020), for the selected four-profile solution, we conducted an additional numerical stability check using 500 initial-stage and 100 final-stage random starts. The best log-likelihood value was replicated in both analyses, and the re-estimated model yielded identical fit indices, class proportions, classification quality, and profile-specific indicator means. Replication of the best log-likelihood value indicated that the solution was stable and likely represented the global maximum. Model fit was evaluated using multiple criteria, including Akaike Information Criterion (AIC), Bayesian Information Criteria (BIC), and sample-size adjusted BIC (SSA-BIC), where lower values indicate better fit (Nylund et al., 2007). The Lo-Mendell-Rubin adjusted likelihood ratio test (LMR-LRT) and the Bootstrap Likelihood Ratio Test (BLRT) were used to compare models with K and K-1 profiles, with significant results indicating improved fit for the model with more profiles. Entropy was examined as an indicator of classification precision, with values above 0.70 considered acceptable (Clark & Muthén, 2009). Model fit indices for all solutions are presented in Table 2.

After identifying the latent profiles, the BCH method was used to examine differences in mental health outcomes across profiles. This approach allows comparisons of mean levels (e.g., subjective well-being, internalizing and externalizing problems) while accounting for classification uncertainty, thereby providing more reliable estimates than methods that assign individuals to their most likely class without adjustment (Bolck et al., 2004; Asparouhov & Muthén, 2014).

## Results

### Descriptive Statistics and Correlations

Descriptive statistics and zero-order correlations are presented in Table 1. Competition and skill development motives were significantly positively associated with subjective well-being and only competition motives were significantly negatively associated with internalizing problems. In contrast, escape, coping, and fantasy motives were significantly negatively associated with well-being and significantly positively associated with both internalizing and externalizing problems. Social motives were significantly positively associated with well-being and significantly negatively associated with externalizing problems, while recreation motives were not significantly associated with the mental health variables. Within gender, girls reported significantly lower well-being and significantly higher levels of internalizing problems, whereas no significant association was observed with externalizing problems.

**Table 1:** Means, standard deviations, ranges, and Pearson correlations among study variables

Variable	*M*	*SD*	Min	Max	1	2	3	4	5	6	7	8	9	10	11
**1. Wellbeing**	26.50	4.60	7	35	-										
**2. Internalizing problems**	8.13	2.62	4	15	− 0.62***	-									
**3. Externalizing problems**	9.55	1.87	3	19	− 0.22***	0.20***	-								
**4. Social**	8.31	4.08	1	20	0.09*	− 0.06	− 0.09*	-							
**5. Escape**	7.61	4.45	1	20	− 0.26***	0.33***	0.18***	0.57***	-						
**6. Competition**	9.48	4.65	1	20	0.12**	− 0.12**	0.09	0.60***	0.40***	-					
**7. Coping**	8.72	3.98	1	20	− 0.09	0.16***	0.11*	0.67***	0.77***	0.58***	-				
**8. Skill development**	8.49	4.67	1	20	0.11*	− 0.07	0.09	0.69***	0.54***	0.64***	0.76***	-			
**9. Fantasy**	7.61	4.28	1	20	− 0.13**	0.13**	0.17***	0.59***	0.76***	0.50***	0.73***	0.65***	-		
**10. Recreation**	13.02	4.31	1	20	0.03	0.04	− 0.02	0.47***	0.44***	0.47***	0.61***	0.57***	0.49***	-	
**11. Gender (0 = boys**,** 1 = girls)**	0.41	0.49	0	1	− 0.31***	0.42***	− 0.06	− 0.36***	− 0.12**	− 0.41***	− 0.23***	− 0.34***	− 0.21***	0.21***	− 0.43***

Note: *p < 0.05 **p < 0.01 ​***p > 0.001

### Identification of Motivational Profiles

The four-profile solution showed acceptable entropy and significant likelihood ratio tests, indicating better fit than the three-profile solution. Although the five-profile solution showed slightly lower information criteria, the additional class represented less than 5% of the sample and closely resembled an existing profile. Given the minimal improvement in fit, small class size, and considerations of parsimony and interpretability, the four-profile solution was retained as the final model (Spurk et al., 2020).

**Table 2:** Model fit statistics from latent profile solutions

Profile solution	AIC	BIC	SSA-BIC	Adj. LMR-LRT (*p*)	BLRT	Entropy
1-class	-	-	-	-	-	-
2-class	17955.549	18047.600	17977.774	1495.213 (0.010)	1474.425	0.930
3-class	17510.622	17636.147	17540.929	451.795 (0.002)	460.927	0.875
4-class	17238.755	17397.753	17277.143	282.164 (0.004)	287.867	0.906
5-class	17063.025	17255.496	17109.495	187.932 (0.038)	191.730	0.900
6-class	16939.261	17165.205	16993.812	136.995 (0.170)	139.764	0.920

Note. AIC = Akaike information criterion; BIC = Bayesian information criterion; SSA-BIC = sample-size-adjusted BIC; LMRT = Lo-Mendell-Rubin adjusted likelihood ratio test test; BLRT = bootstrapped log-likelihood ratio test. Lower AIC, BIC, and SSA-BIC values indicate better model fit. Higher entropy values indicate greater classification accuracy

**Fig. 1:**
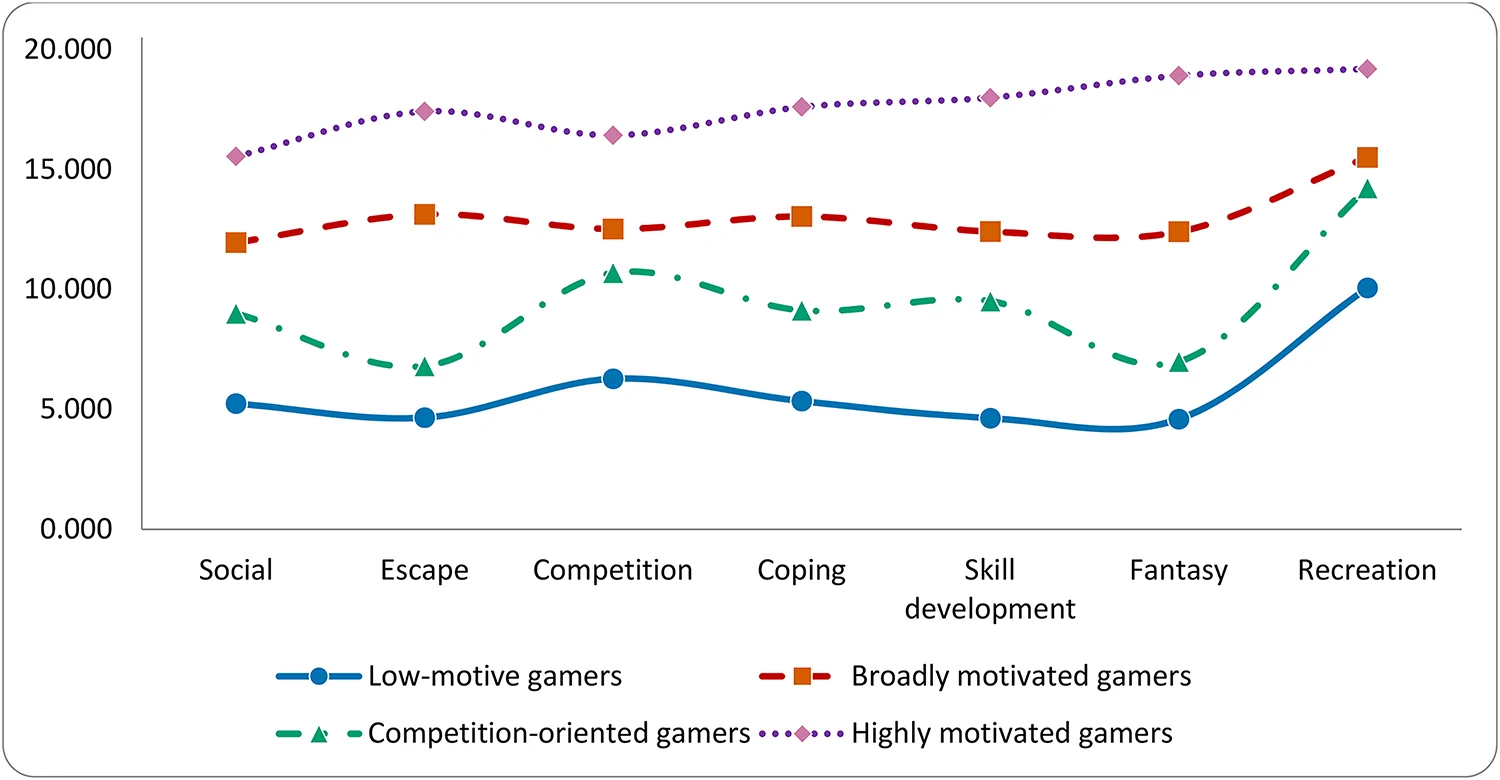
Four-class latent profile solution of gaming motives. Note. The four-class solution consisted of four profiles: *Profile 1 (Low-motive gamers, 25.3% n = 171) **Profile 2 (broadly motivated gamers, 41.5% n = 201), ***Profile 3 (Competition-oriented gamers, 18.6%, n = 90), and ****Profile 4 (highly motivated gamers, 4.5%, n = 22). Values represent profile-specific estimated summed means for each MOGQ motive derived from the latent profile analysis

The four-profile solution is illustrated in Fig. 1. Profile 1 (Low-motive gamers) showed generally low levels across all motives. Profile 2 (Broadly motivated gamers) displayed broad and moderate levels across motives. Profile 3 (Competition-oriented gamers) was characterized by relatively higher competition motives compared to other motives. Finally, profile 4 (Highly motivated gamers) showed consistently high levels across all motives, with particularly elevated escape and coping motives. To assess numerical stability, the selected four-profile solution was re-estimated using an increased number of random starts. This analysis reproduced the same solution, including the highly motivated profile, which comprised 4.55% of the analytic sample (*n* = 22); classification quality also remained high (entropy = 0.907).

### Differences in Adolescents’ Mental Health Across Motivational Profiles

The BCH analysis indicated significant differences between the latent profiles in externalizing problems (χ² = 64.01, *p* < .001), internalizing problems (χ² = 45.29, *p* < .001), and subjective well-being (χ² = 34.63, *p* < .001). As shown in Table 3, broadly motivated gamers reported the highest levels of internalizing problems and the lowest well-being, whereas highly motivated gamers reported the highest levels of externalizing problems. In contrast, broadly motivated gamers also showed the lowest levels of externalizing problems.

**Table 3:** Means of mental health outcomes across latent profiles

Profile	Externalizing M (SE)	Internalizing M (SE)	Well-being M (SE)
Low motive gamers	5.34 (0.30)	6.23 (0.92)	20.93 (1.07)
Broadly motivated gamers	2.73 (0.26)	14.49 (0.96)	12.22 (1.07)
Competition-oriented gamers	5.54 (0.45)	6.93 (1.18)	18.81 (1.40)
Highly motivated gamers	7.02 (0.93)	4.86 (2.40)	20.54 (2.35)

Note. Values represent BCH-adjusted estimated means with standard errors in parentheses

## Discussion

Digital gaming constitutes a central part of many adolescents’ everyday lives, and its implications for mental health have received increasing attention in recent decades. Although previous research has often focused on the amount of time adolescents spend gaming (Ballou et al., 2025), accumulating evidence suggests that gaming duration alone shows relatively weak and inconsistent associations with psychological outcomes (Ballou et al., 2025; Király et al., 2017). This has led to growing interest in understanding the psychological motivations underlying gaming behavior. Drawing on Uses and Gratification Theory (UGT) (Katz et al., 1973), gaming behavior can be understood as goal-directed, where individuals actively engage with gaming to satisfy specific psychological and social needs (Katz et al., 1973). From this perspective, gaming motivations may help explain why gaming is associated with psychopathology and subjective well-being. The current study contributes to this line of research by identifying distinct motivational profiles among adolescent gamers and examining how these profiles differ with respect to internalizing and externalizing problems as well as subjective well-being. In short, the study found four distinct profiles of adolescents characterized by various degrees of gaming motivations and that some of these profiles were differentially related to mental health functioning in adolescents.

### Gaming Motivational Profiles

Building on prior research suggesting that gaming motivations cluster into distinct patterns (e.g., Meriläinen et al., 2023), the current study identified four motivational profiles: low-motive gamers, broadly motivated gamers, competition-oriented gamers, and highly motivated gamers. Low-motive gamers reported low levels across all motives, which may suggest a limited engagement with gaming in a way that is specifically driven by the motivations assessed. This profile is comparable to the lowly-motivated casual gamer profile identified by Kim et al. (2022). Competition-oriented gamers in this study were characterized by higher levels of competition and recreation motives and relatively low escape and coping motives, suggesting that gaming for this group may function primarily as a domain of challenge, mastery, and enjoyment rather than avoidance (Kaye et al., 2017). This profile aligns closely with the achiever and recreational profiles identified in prior longitudinal research (Meriläinen et al., 2023; Wang et al., 2021).

In contrast, both the broadly motivated and highly motivated profiles were characterized by elevated escape and coping motives alongside other gaming motives, suggesting that gaming for these groups may serve avoidance-oriented functions such as emotion regulation and stress management (Bäcklund et al., 2025; Hellström et al., 2015; King & Delfabbro, 2020). While highly motivated gamers showed the most pronounced elevation across all motives — particularly escape and coping — broadly motivated gamers displayed a similarly elevated avoidance-oriented pattern at a somewhat lower overall intensity. Together, these two profiles most closely resemble the escapist profile described by Meriläinen et al. (2023) and the highly motivated-dissatisfied profile identified by Kim et al. (2022), both of which were associated with the most adverse psychological outcomes.

### Motivational Profiles and Adolescent Mental Health

The four motivational profiles differed significantly in internalizing problems, externalizing problems, and subjective well-being, highlighting the multidimensional nature of mental health in adolescence (Plenty et al., 2021). Importantly, higher levels of gaming motivation were associated with distinct patterns of mental health indicators rather than a single form of psychological risk.

As expected, among the two avoidance-oriented profiles, broadly motivated gamers reported the highest levels of internalizing problems and the lowest levels of well-being compared to normative Swedish adolescent population levels (Public Health Agency of Sweden, 2018), indicating a potentially vulnerable group. This is consistent with prior research linking escape and coping motives to depressive symptoms and psychological distress (Hellström et al., 2015; Wartberg et al., 2017), and mirrors the adverse outcomes associated with the escapist profile in Meriläinen et al. (2023) and the highly motivated-dissatisfied profile in Kim et al. (2022). As hypothesized, highly motivated gamers, the second avoidance-oriented profile, reported the highest levels of externalizing problems, alongside lower internalizing problems and somewhat unexpectedly, relatively high well-being. Compared with the broadly motivated profile, this group reported relatively higher fantasy and skill-development motives, suggesting a greater relative emphasis on the immersive, stimulating, and challenging aspects of gaming. One tentative interpretation is that individual characteristics such as sensation-seeking or impulsivity may be relevant to both this pattern of gaming motivation and externalizing difficulties (Johannes et al., 2021), particularly because games can provide rapid feedback, challenge, and reward (Koivisto & Hamari, 2019). The pattern may also be relevant to adolescents with attention-related difficulties, who may be particularly attracted to highly stimulating yet structured digital environments (Bäcklund et al., 2025). However, these explanations were not directly tested, and the cross-sectional design precludes conclusions about mechanisms or temporal direction, warranting further longitudinal research.

Among the two engagement-oriented profiles, competition-oriented gamers showed comparatively favorable psychological outcomes, including relatively high well-being and lower internalizing problems, with well-being levels broadly consistent with normative Swedish adolescent population levels (Public Health Agency of Sweden, 2018). This pattern suggests that gaming motivated primarily by competition and skill development may be associated with a relatively adaptive developmental trajectory (Collado-Soler et al., 2023), which aligns with the favorable outcomes associated with achiever and recreational profiles in prior research (Meriläinen et al., 2023; Wang et al., 2021). Finally, low-motive gamers displayed relatively moderate levels across all mental health outcomes, suggesting that gaming may function as a casual leisure activity for this group rather than a psychologically or developmentally significant behavior.

The results point to the importance of underlying motivational patterns in understanding adolescents’ psychopathology and subjective well-being, consistent with previous research (e.g., Gisbert-Pérez et al., 2024). Seen in this light, gaming may be viewed less as a risk factor and more a window into adolescents’ emotional and motivational lives. Understanding adolescents’ reasons for gaming may therefore contribute to identifying both patterns associated with vulnerability to psychopathology and adaptive forms of engagement, providing a clearer understanding of the role digital gaming in adolescent development. The current study extends prior work by demonstrating that these motivational configurations are not merely descriptive but systematically associated to differential patterns of psychopathology and well-being.

### Limitations and Strengths of the Study

Several methodological limitations warrant consideration. First, all key constructs relied on self-report which may introduce common method bias and shared method variance and inflate associations between variables (Polas, 2025). Although self-report measures are appropriate for assessing internal states such as motivations and well-being, future research could strengthen measurement validity by incorporating multi-informant data, such as parent or teacher reports, as well as behavioral gaming logs (Parry et al., 2021). Furthermore, the analytic sample may not fully represent the broader adolescent population, as excluded participants were more often girls and adolescents with migrant backgrounds, which may influence the generalizability. Another limitation concerns the psychometric performance of some of the measures. The externalizing-problems scale initially demonstrated low internal consistency, although reliability improved after removing one item with a weak item–total correlation (Hair et al., 2019). In addition, while the items showed satisfactory loadings on their respective MOGQ dimensions, the mixed overall model fit and high correlations among several latent factors suggested limited discriminant separation between some dimensions. Further psychometric research using independent samples of gamers and alternative measurement models is therefore needed to clarify the structure, reliability, and validity of the MOGQ. Finally, the latent profile analysis (LPA), where the highly motivated gamer profile represented a relatively small subgroup within the four-profile solution. Although small latent profiles may raise concerns about statistical stability and generalizability (Sinha et al., 2021), mixture modeling research suggests that rare profiles should not be dismissed if they reflect theoretically meaningful patterns (Spurk et al., 2020). An alternative would have been to retain the five-profile solution, as both the four- and five-profile models included a class below 5%. However, the five profile-solution did not yield a substantively distinct additional profile and showed lower statistical stability. In contrast, the four-profile solution demonstrated stronger stability and clearer differentiation and is also consistent with prior research in the field that has identified three- to four-profile solutions (e.g., Meriläinen et al., 2023).

Despite these limitations, the current study has several important methodological strengths. The combination of variable-centered and person-centered analytical approaches allowed for a more comprehensive examination of gaming motivations than either method alone would permit. Additionally, the use of the well-established Motives for Online Gaming Questionnaire (MOGQ; Demetrovics et al., 2011) strengthens confidence in the validity of the motivational constructs assessed (Wen et al., 2025). In addition, the study assessed both mental health problems and positive well-being, which aligns with contemporary dual-continua models of mental health (Keyes, 2005). A further methodological strength is the use of the BCH method for distal outcome comparisons. Traditional approaches often involve assigning individuals to their most likely latent class and subsequently comparing these groups using conventional statistical methods such as analysis of variance or regression analyses (Spurk et al., 2020). However, such because latent profiles represent probabilistic rather than directly observed group membership, conventional approaches may produce biased estimates by ignoring classification uncertainty (Bakk & Kuha, 2021). The BCH approach addresses this limitation by preserving the latent profile solution while accounting for uncertainty in class assignment, thereby strengthening the validity of comparisons between profiles (Asparouhov & Muthén, 2014; Bakk & Kuha, 2021).

### Implications and Future Directions

The present findings carry several implications for both research and practice. Most fundamentally, they suggest that the psychological motivations underlying gaming provide a more meaningful lens for understanding adolescent psychopathology and subjective well-being than a focus on gaming behavior alone. Much public discourse and policy discussion treats gaming primarily as a question of exposure, yet the current results indicate that why adolescents game — and what psychological needs gaming fulfills — may be more informative than how much they game. This distinction has direct practical relevance. Rather than screening solely for symptoms of problematic gaming, practitioners may benefit from also assessing adolescents’ underlying gaming motives. Escape- or coping-driven gaming may signal underlying emotional distress and emerging psychopathology and could serve as a useful indicator for identifying adolescents who may benefit from additional psychological support, whereas gaming motivated by competition or mastery may reflect more structured and developmentally adaptive engagement with digital environments.

Future research should examine motivational patterns more systematically, particularly through longitudinal designs that can illuminate how gaming motives develop across adolescence and how they relate to developmental trajectories of both psychopathology and subjective well-being. Gender differences in gaming motivations and associated mental health outcomes also warrant further investigation, given that boys and girls often display distinct motivational preferences and patterns of gaming engagement. Finally, future studies should examine whether different gaming contexts — such as online versus offline or different game genres — are associated with distinct motivational profiles and differential mental health outcomes, as the present study did not differentiate between these forms of engagement.

## Conclusion

Although digital gaming is central to many adolescents’ everyday lives, research has provided limited understanding of how the psychological motives underlying gaming co-occur within individuals and relate to both psychopathology and subjective well-being. The present study addressed this gap using a person-centered approach to examine gaming motives and their associations with adolescent psychopathology and well-being. Latent profile analysis identified four distinct motivational profiles: low-motive, broadly motivated, competition-oriented, and highly motivated gamers. Broadly motivated gamers reported the highest levels of internalizing problems and the lowest well-being, whereas highly motivated gamers reported the highest levels of externalizing problems despite relatively high well-being. These findings show that gaming is not a uniform developmental experience; rather, its psychological significance depends on the constellation of motives it serves. By identifying motivational patterns associated with both vulnerability to psychopathology and more adaptive functioning, the study advances understanding of gaming as a meaningful context for adolescent emotional, behavioral, and social development.

## Supplementary Information

Below is the link to the electronic supplementary material.

Supplementary Material 1

## Data Availability

The pseudonymized data and analysis codes supporting the findings of this study have been made openly available on the Open Science Framework (OSF) and can be accessed at https://osf.io/xpmnr/ (view-only link: https://osf.io/xpmnr/?view_only=71bf43091fa84340ad7f4da4df949fdc). The data are pseudonymized in accordance with ethical and privacy requirements.
