# Supplementary material for: Motivational Profiles of Adolescent Gaming and Their Associations with Psychopathology and Well-Being
Source: J Youth Adolesc. 2026 Sep 24;55(11):2751–63. doi: 10.1007/s10964-026-02414-w (PMC13768278; doi:10.1007/s10964-026-02414-w)
Supplement: Supplementary file 1 [file 10964_2026_2414_MOESM1_ESM.docx]

**Motivational Profiles of Adolescent Gaming and Their Associations with Psychopathology and Well-Being**

Table S1

Standardized Item Factor Loadings for the MOGQ

| Motive | Item | Standardized loading |
| --- | --- | --- |
| Social | T1_V13_MOGQ1 | 0.635 |
| Social | T1_V13_MOGQ8 | 0.711 |
| Social | T1_V13_MOGQ15 | 0.800 |
| Social | T1_V13_MOGQ22 | 0.769 |
| Escape | T1_V13_MOGQ2 | 0.817 |
| Escape | T1_V13_MOGQ9 | 0.899 |
| Escape | T1_V13_MOGQ16 | 0.914 |
| Escape | T1_V13_MOGQ23 | 0.829 |
| Competition | T1_V13_MOGQ3 | 0.674 |
| Competition | T1_V13_MOGQ10 | 0.712 |
| Competition | T1_V13_MOGQ17 | 0.857 |
| Competition | T1_V13_MOGQ24 | 0.883 |
| Coping | T1_V13_MOGQ4 | 0.650 |
| Coping | T1_V13_MOGQ11 | 0.764 |
| Coping | T1_V13_MOGQ18 | 0.636 |
| Coping | T1_V13_MOGQ25 | 0.812 |
| Skill Development | T1_V13_MOGQ5 | 0.876 |
| Skill Development | T1_V13_MOGQ12 | 0.868 |
| Skill Development | T1_V13_MOGQ19 | 0.857 |
| Skill Development | T1_V13_MOGQ26 | 0.889 |
| Fantasy | T1_V13_MOGQ6 | 0.685 |
| Fantasy | T1_V13_MOGQ13 | 0.815 |
| Fantasy | T1_V13_MOGQ20 | 0.845 |
| Fantasy | T1_V13_MOGQ27 | 0.855 |
| Recreation | T1_V13_MOGQ7 | 0.558 |
| Recreation | T1_V13_MOGQ14 | 0.600 |
| Recreation | T1_V13_MOGQ21 | 0.670 |
| Recreation | T1_V13_MOGQ28 | 0.823 |

*Note*. Values are standardized loadings and significant at p < .001. The revised model included the following standardized within-factor residual correlations: T1_V13_MOGQ1 with T1_V13_MOGQ8, r = .518; T1_V13_MOGQ3 with T1_V13_MOGQ10, r = .501; T1_V13_MOGQ13 with T1_V13_MOGQ20, r = .445; and T1_V13_MOGQ14 with T1_V13_MOGQ21, r = .393 (all ps < .001).

Table S2

Standardized Correlations Among the MOGQ Latent Factors

| Motive | 1 | 2 | 3 | 4 | 5 | 6 | 7 |
| --- | --- | --- | --- | --- | --- | --- | --- |
| Social | — | — | — | — | — | — | — |
| Escape | 0.636 | — | — | — | — | — | — |
| Competition | 0.696 | 0.494 | — | — | — | — | — |
| Coping | 0.822 | 0.888 | 0.694 | — | — | — | — |
| Skill Development | 0.822 | 0.565 | 0.712 | 0.855 | — | — | — |
| Fantasy | 0.702 | 0.873 | 0.620 | 0.895 | 0.713 | — | — |
| Recreation | 0.638 | 0.552 | 0.552 | 0.785 | 0.665 | 0.629 | — |

Note. 1 = Social; 2 = Escape; 3 = Competition; 4 = Coping; 5 = Skill Development; 6 = Fantasy; 7 = Recreation; All correlations were significant at p < .001.
